# Remarks on the Beneficial Effects of Compression in Rheumatism, as Recommended by Dr. Balfour; with a Case

**Published:** 1815-09

**Authors:** 


*For the London Medical and Physical Journal.*

### *Remarks on the beneficial Effects of Compression in Rheumatism, as recommended by Dr. Balfour; with a Case.*

HAVING observed, in your Critical Analysis for July, the brief notice of a paper contained in the Edinburgh Journal for April, on Rheumatism, by Dr. Balfour, I wish to excite particular attention thereto, through the medium of your widely-circulating Journal, as replete with many valuable practical remarks, and as recommending a new and apparently superior mode of treating an excruciating painful disease, alike relieving the torments attending its acute form, and, in many cases, preventing the pain and inconveniences inseparable from its chronic state. The treatment consists in the application of pressure to the inflamed part, conjoined with the antiphlogistic plan.

Since

Since my perusal of Dr. Balfour's cases, I have had an opportunity of making trial of compression, in two cases, with the most complete success, which I here subjoin, in confirmation of the practice recommended by the respectable originator.

April 16th, I was requested to visit a young woman, aged 25, on account of acute rheumatism. I found her seated, with her right leg placed upon a chair; and learnt the disease was seated in that ankle, which was extremely red, very hot and painful, and much swollen. On her attempting to put her foot to the ground, at my request, she was forced to scream, from the violence of the pain it occasioned, and could not do it. Tongue coated; pulse 110; skin hot. I made a gentle pressure upon the inflamed part with my hand, which gave much pain; but, as I increased the pressure, the pain diminished; and, when firm and strong, she said the part was altogether easy, as Dr. Balfour has related in a similar case. Thinking this a favourable case for the trial of a bandage, I immediately applied a roller *firmly*, around the ancle, commencing from the toes, and extended upwards to the knee. I then requested her walking up stairs to her bed-room (three stories high): she smiled, and said, you know I cannot bear it upon the ground. I told her to try again; and she went up stairs, lame, certainly, but with a firm step, and did not appear fearful of bearing fully upon it. Complains also of some pain in the right knee, and in her back. I gave her Magnes. Sulph. cū Infus. Sennæ; and left directions that she might undertake her usual routine of employment the next morning (being a servant), if she felt able and disposed. Nevertheless, I confess I was astonished, on repeating my visit next day (April 17th), to find the door opened to me by my patient, who had arisen at her usual time, and performed her domestic duties. Has had a good night; tongue cleaner; pulse 90, and softer; ankle nearly free from pain; the opening medicine has procured four stools; no increase of pain in the knee or back; ordered a saline mixture. April 18th, 10 o'clock A. M. has had a return of pain in the last two hours, which I found to originate from the bandage being loose. The redness and swelling nearly gone; pulse 80; tongue moist; re applied the roller; pain in the knee and back gone. The next day, all appearance and feeling of the disease had disappeared. She continued to wear the bandage a week, and then discontinued it. This woman was confined three weeks, by the same disease, six months prior to the afore-mentioned attack, under the usual remedies.

The second case was in every way similar to the above, excepting excepting the site of the disease being in the wrist, and its giving way rather more tardily.

With a conviction of the utility attached to a collection of facts, in medicine or surgery, towards the perfection of any branch thereof, and science in general, and the consequent contribution to the lives and comforts of our fellow beings, I submit the fore-written to my medical brethren, with a hope that a satisfactory inquiry into the modus operandi may result, and an enlightened view taken therefrom, of the yet disputed proximate cause and seat of this painful and frequent disease, which is so essential towards the proper application of remedies.

*August* 3, 1815. OBSERVER.

